# Dataset of the human homologues and orthologues of lipid-metabolic genes identified as DAF-16 targets their roles in lipid and energy metabolism

**DOI:** 10.1016/j.dib.2017.02.055

**Published:** 2017-03-08

**Authors:** Lavender Yuen-Nam Fan, Paula Saavedra-García, Eric Wing-Fai Lam

**Affiliations:** Department of Surgery and Cancer, Imperial College London, Hammersmith Hospital Campus, London W12 0NN, UK

**Keywords:** ACAA2, Acetyl-CoA acetyltransferase 2, ACACA, Acetyl-CoA carboxylase, ACAD8, Acyl-CoA dehydrogenase family member 8, ACADM, Acyl-CoA dehydrogenase C4 to C12 straight chain, ACOX, Acyl-CoA oxidase, ACSL3/4, Acyl-CoA synthetase long-chain family member 3/4, ACSS, Acyl-CoA synthetase short-chain family member, CPT2, Carnitine palmitoyltransferase II, DAG, Diacylglycerol, DGAT, Diacylglycerol O-acyltransferase, ECHS1, Short-chain enoyl-CoA hydratase 1, ELOVL1, Elongation of very long chain fatty acids protein 1, FA, fatty acid, FADS2, Fatty acid desaturase 2, FASN, Fatty acid synthase, FATP4, Fatty acid transport protein 4, FOX, Forkhead box, HADH, Hydroxyacyl-coenzyme A dehydrogenase, HADHA, Hydroxyacyl-CoA dehydrogenase/3-Ketoacyl-CoA thiolase/Enoyl-CoA hydratase, alpha subunit, LCFA, Long chain fatty acid, MLYCD, Malonyl-CoA decarboxylase, MOGAT1/2, Monoacylglycerol O-acyltransferase 1/2, PNPLA, patatin like phospholipase domain containing, PUFA, polyunsaturated fatty acid, SCD1/5, Stearoyl-CoA desaturase 1/5, TAG, triacylglycerol, TCA, Tricarboxylic acid, VLCFA, Very long chain fatty acid., FOXO3, FOXM1, DAF-16, *Caenorhabditis elegans*, Lipid metabolism

## Abstract

The data presented in this article are related to the review article entitled ‘Unravelling the role of fatty acid metabolism in cancer through the FOXO3-FOXM1 axis’ (Saavedra-Garcia et al., 2017) [24]. Here, we have matched the DAF-16/FOXO3 downstream genes with their respective human orthologues and reviewed the roles of these targeted genes in FA metabolism. The list of genes listed in this article are precisely selected from literature reviews based on their functions in mammalian FA metabolism. The nematode *Caenorhabditis elegans* gene orthologues of the genes are obtained from WormBase, the online biological database of *C. elegans.* This dataset has not been uploaded to a public repository yet.

**Specifications Table**

**Table:** 

Subject area	Cell Biology
More specific subject area	Lipid metabolism
Type of data	Table
How data was acquired	Online database, Literature Review
Data format	Review
Experimental factors	
Experimental features	
Data source location	Online database
Data accessibility	Within this article

**Value of data**

• The dataset shows the extensive overlap in the signaling pathway and molecules involved in the FOXO3-FOXM1 axis and FA metabolism. The assessment of this interaction can be of value for research group from related fields.
• The dataset allows integration of researches in oncology and metabolic diseases.
• The dataset opens up new approaches to study the roles of FOXO3-FOXM1 axis and FA metabolism in breast cancer initiation, progression and metastasis and drug resistance.
• These data also be useful for researches in all cancer types where lipid and energy metabolism is implicated.

## 1 Data

Based on literature reviews, we observed that the DAF-16 is the *C. elegans* orthologues of mammalian forkhead box (FOX) proteins, FOXO3 and FOXM1, and also the primary factor essential for enhancing the expression of gene networks involved in regulation of fatty acid (FA) metabolism [1], [5], [9], [24], [25]. The dataset of this article provides information of the genes that are overlapped in cellular pathways involved in both FOXO3-FOXM1 axis and FA metabolism [4], [13], [18], [35]. From Amrit *et al.*, we selected a list of FA metabolism regulatory genes that are downstream of DAF-16/FOXO3 [1]. To better understand the roles of FOXO3-FOXM1 axis in FA metabolism, we then matched the genes with their respective human orthologues using WormBase (www.wormbase.org), and their roles in lipid and energy metabolism are summarized in Table 1.

## Figures and Tables

**Table 1:** List of Human homologues and orthologues of lipid-metabolic genes identified as DAF-16 and/or TCER-1 targets through RNA-Seq by [1] and their potential functions in lipid metabolism.

**C.*****elegans*****genes regulated by DAF-16/FOXO3**	**Human orthologues (from WormBase)**[27]	**Role in lipid and energy metabolism**
*pod-2*	Acetyl-CoA carboxylase alpha (ACACA)	The major regulatory enzyme involved in rate-limiting step of *de novo* FA biosynthesis [11]. ACACA promotes FA biosynthesis by catalyzing the conversion of acetyl-CoA to Malonyl-CoA, a building block of FA [11].
*mlcd-1*	Malonyl-CoA decarboxylase (MLYCD)	An enzyme that produces acetyl-CoA from malonyl-CoA [14], thus increase the rate of β-oxidation.
*fasn-1*	Fatty acid synthase (FASN)	The major regulatory enzyme that mediates the *de novo* synthesis of saturated FAs from acetyl-CoA and malonyl-CoA [2].
*mboa-2*	Diacylglycerol O-acyltransferase (DGAT) 1	One of 2 enzymes that catalyze the final step in triacylglycerol (TAG) synthesis in which diacylglycerol (DAG) is covalently bound to long chain fatty acyl-CoAs [26].
*dgat-2*	DGAT2	The second enzymes that catalyze the final reaction in the synthesis of TAG [28].
*acs-22*	Fatty acid transport protein 4 (FATP4)	The membrane transporter essential for translocation of very long chain fatty acid (VLCFA) across the plasma membrane into cells [6].
*Y53G8B.2, K07B1.4*	Monoacylglycerol O-acyltransferase 1/2 (MOGAT1/2), DGAT2	Enzymes predicted to have transferase activity, transferring acyl groups other than amino-acyl groups [28] and catalyze the synthesis of DAGs, precursor of TAGs and phospholipids [34].
*fat-5, fat-6, fat-7*	Stearoyl-CoA desaturase 1/5 (SCD1/5)	Integral membrane proteins of the endoplasmic reticulum that catalyzes the formation of monounsaturated FAs from saturated FAs, a key regulator of energy metabolism [30].
*lipl-1, lipl-2, lipl-5*	Lipase A (orthologue of *lipl-1*), Lipase F, gastric type (orthologue of *lipl-1* and *lipl-2*), and other members of the Lipases family	Proteins involved in lipid (cholesteryl ester) storage; *lipl-1 and lipl-2* are predicted to have hydrolase activity, acting on ester bonds of cholesteryl esters and TAGs [23].
*atgl-1*	Patatin like phospholipase domain containing (PNPLA) 1, PNPLA2 and PNPLA3	Proteins that have diverse lipolytic and acyltransferase activities and are key elements in lipid metabolism [12].
*acs-17*	Acyl-CoA synthetase long-chain family member 3/4 (ACSL3/4)	Long-chain acyl-CoA synthetases, responsible for convert long chain fatty acids (LCFAs) into fatty acyl-CoA in order for it to enter the mitochondria and to be β-oxidized [3].
*cpt-2*	Carnitine palmitoyltransferase II (CPT2)	Transporter protein at mitochondrial inner member that catalyzes an acyl-group transfer between added CoA and carnitine [32], thus facilitates β-oxidation pathway in the mitochondria.
*acdh-9*	Acyl-CoA dehydrogenase family, member 8 (ACAD8)	A mitochondrial enzyme catalyzes the alpha, beta-dehydrogenation of acyl-CoA derivatives in the metabolism of FAs or branch chained amino acid [20], [29]
*ech-7*	Short-chain enoyl-CoA hydratase (ECHS1)	Enzyme functions in the second step of the mitochondrial β-oxidation pathway. It catalyzes the hydration of 2-trans-enoyl-coenzyme A intermediates to L-3-hydroxyacyl-CoAs [8].
*ech-1.2*	Hydroxyacyl-CoA	The tri-functional protein that catalyzes the last three steps of mitochondrial β-oxidation of LCFAs [22].
	Dehydrogenase/3-Ketoacyl-CoA	
	Thiolase/Enoyl-CoA Hydratase, Alpha Subunit (HADHA)	
*acox-1*	Acyl-CoA oxidase 1 (ACOX1)	The enzyme catalyzes the first, rate-limiting step in peroxisomal β-oxidation of medium to very long straight-chain FA [17].
*F08A8.2, F08A8.3, F08A8.4, C48B4.1*	ACOX2	The key regulatory enzyme of the β-oxidation pathway 2 for side chain oxidation of cholesterol [15], [33].
*acaa-2*	Acetyl-CoA acetyltransferase 2(ACAA2)	An enzyme involved in the last step of mitochondrial β-oxidation [19].
*hacd-1*	Hydroxyacyl-coenzyme A dehydrogenase (HADH)	An enzyme functions in the mitochondrial matrix to catalyze the oxidation of straight-chain 3-hydroxyacyl-CoAs as part of the β-oxidation pathway. Its enzymatic activity is higher with medium-chain-length FAs [31].
*fat-2*	Fatty acid desaturase 2(FADS2)	A delta-12 fatty acyl desaturase that catalyzes the formation of double bond between defined carbons of fatty acyl chain [10].
*acs-2*	Members of Acyl-CoA synthetase family, such as acyl-CoA Synthetase Short-Chain Family member (ACSS) 1, ACSS2 and ACSS3	Enzymes predicted to catalyze the conversion of acetate to acetyl-CoA in order for it to feed to the tricarboxylic acid (TCA) cycle to produce ATP [27].
*elo-1, elo-2*	Polyunsaturated fatty acid (PUFA) elongases, such as Elongation of very long chain fatty acids protein 1 (ELOVL1)	Microsomal enzymes involved in VLCFA elongation during increased β-oxidation [7], [21]
*acdh-11*	Acyl-CoA dehydrogenase family members, such as Acetyl-CoA Dehydrogenase C4 to C12 Straight Chain (ACADM)	Very-long-chain specific acyl-CoA dehydrogenase that catalyze the initial step of the mitochondrial β-oxidation, specific to C4 to C12 straight chain [16].
*lips-10, lips-14*	LIPase related protein	Pseudogenes of *lipl-1*, *-2* and *-5*, proteins predicted to have hydrolase and lipid storage activity [27].
